# Impact of a borderless sample transport network for scaling up viral load monitoring: results of a geospatial optimization model for Zambia

**DOI:** 10.1002/jia2.25206

**Published:** 2018-12-04

**Authors:** Brooke E Nichols, Sarah J Girdwood, Thomas Crompton, Lynsey Stewart‐Isherwood, Leigh Berrie, Dorman Chimhamhiwa, Crispin Moyo, John Kuehnle, Wendy Stevens, Sydney Rosen, EQUIP Health

**Affiliations:** ^1^ Department of Global Health School of Public Health Boston University Boston MA USA; ^2^ Health Economics and Epidemiology Research Office Department of Internal Medicine School of Clinical Medicine Faculty of Health Sciences University of the Witwatersrand Johannesburg South Africa; ^3^ GIS Mapping Department Right to Care Centurion South Africa; ^4^ National Health Laboratory Service Wits Medical School Johannesburg South Africa; ^5^ EQUIP Zambia Lusaka Zambia; ^6^ Department of Molecular Medicine and Haematology Faculty of Health Sciences University of the Witwatersrand Johannesburg South Africa; ^7^ United States Agency for International Development Lusaka Zambia

**Keywords:** viral load scale‐up, patient monitoring, geospatial modelling, cost modelling, health systems, viral load

## Abstract

**Introduction:**

The World Health Organization recommends viral load (VL) monitoring at six and twelve months and then annually after initiating antiretroviral treatment for HIV. In many African countries, expansion of VL testing has been slow due to a lack of efficient blood sample transportation networks (STN). To assist Zambia in scaling up testing capacity, we modelled an optimal STN to minimize the cost of a national VL STN.

**Methods:**

The model optimizes a STN in Zambia for the anticipated 1.5 million VL tests that will be needed in 2020, taking into account geography, district political boundaries, and road, laboratory and facility infrastructure. We evaluated all‐inclusive STN costs of two alternative scenarios: (1) optimized status quo: each district provides its own weekly or daily sample transport; and (2) optimized borderless STN: ignores district boundaries, provides weekly or daily sample transport, and reaches all Scenario 1 facilities.

**Results:**

Under both scenarios, VL testing coverage would increase to from 10% in 2016 to 91% in 2020. The mean transport cost per VL in Scenario 2 was $2.11 per test (SD $0.28), 52% less than the mean cost/test in Scenario 1, $4.37 (SD $0.69), comprising 10% and 19% of the cost of a VL respectively.

**Conclusions:**

An efficient STN that optimizes sample transport on the basis of geography and test volume, rather than political boundaries, can cut the cost of sample transport by more than half, providing a cost savings opportunity for countries that face significant resource constraints.

## 1 Introduction

In order to achieve UNAIDS’ target that 90% of patients who are on antiretroviral therapy (ART) for HIV be virally suppressed, viral load (VL) monitoring at six and twelve months after initiating ART and annually thereafter has been recommended by the World Health Organization as the best way to monitor patients on ART 1. VL monitoring has been shown to be a cost‐effective monitoring strategy for identifying virologic failure due to poor adherence and/or drug resistance 2, 3. With the rapidly increasing number of patients on ART worldwide, establishing accessible and affordable VL monitoring capacity is essential.

Until recently, many low‐ and middle‐income countries did not provide routine VL testing due to high costs, a lack of specialized laboratory facilities and coordinated blood plasma sample transport networks 4. It has been estimated that sample transportation comprises up to a third of the total cost of providing VL testing when non‐optimized sample transportation is used 5. In many instances, the lack of funding for capital purchases (vehicles) or failure to create a sample transport system are greater barriers than the transport cost per test. Countries will require both investment in laboratory capacity and a reliable sample transportation network (STN) to carry blood samples from local clinics to the laboratories.

Zambia, a lower middle‐income country in southern Africa, is one of the countries facing this challenge. An estimated 1.2 million people are infected with HIV in Zambia, of whom just under 800,000 are on ART, of which approximately 40% had access to routine VL testing 6, 7, 8. The Zambian Ministry of Health has set a goal of providing routine testing to 80% of these patients by 2020 6. Rapid, massive scale‐up of VL testing is thus needed.

Zambia has approximately 1500 clinics and hospitals that provide ART. VL testing is currently highly centralized at 19 laboratories; point‐of‐care and near‐point‐of‐care equipment is not yet being considered for use in the Zambian VL scale‐up plan. These 19 centralized laboratories provide VL testing capacity across the country, thus requiring a vast STN for blood samples to move from the clinics to the laboratories. Currently, samples are transported largely on an ad hoc basis. Many districts independently provide sample transport for facilities within their boundaries, and many facilities are responsible for providing their own sample transport using their own vehicles, motorbikes or public transport. Efforts to date have focused on optimizing sample transport within administrative districts, a reflection of both national governance structures and the approach of international donors in which different “implementing partners” have responsibility for supporting different districts. Existing laboratories may be nearer to clinics in adjacent districts, however, hampering efficient sample transport. Broad coordination between all partners is a crucial step towards a sustainable laboratory programme 9.

To assist Zambia in scaling up VL testing capacity, we designed an innovative geospatial optimization model that aimed to minimize the cost of a national VL STN.

## 2 Methods

We developed a geospatial model that utilized a range of existing and new data in order to minimize transport distances and driving times, numbers of vehicles and costs required for VL sample transport. Layers of information were combined in the Environmental Systems Research Institute's (ESRI) software, *ArcGIS 10*.5, a geospatial processing program for geospatial data, such that all parameters could be utilized in the STN optimization based on anticipated 2020 VL sample volumes. The final geospatial model output was then included in a cost model to determine the total cost associated with each scenario. The objective of our model was to maximize coverage of the STN while minimizing the cost of the STN.

### 2.1 Data sources

The sources of information incorporated into the model were largely sourced through EQUIP partners in Zambia. EQUIP is a consortium led by five Africa‐based organizations that provides technical assistance to local governments and implementation partners with the aim of strengthening the HIV treatment and prevention response. Data sources included:

1. Geographical Information System (GIS) facility assessments: more than 2500 healthcare facilities in Zambia were visited by an EQUIP GIS team. A mobile‐based application was developed to facilitate data collection on the Global Positioning System (GPS) location of each facility and basic infrastructure available and to determine current access to VL sample transportation at each facility (including frequency and type of transport). The GPS tracking data from each vehicle was average and added to a routable road layer to determine the accessibility and drive times between all facilities and VL laboratories. This exercise was conducted during the rainy season, when the roads are at their worst condition. This information was used to supplement an existing routable road layer for Zambia 10. Data from the 1484 HIV treatment facilities were used in the final analysis, along with the complete road layer.
2. Laboratory assessments: more than 650 existing laboratories, ranging from very small, low‐technology sites designed to fully equipped centralized laboratories, were assessed by EQUIP using an electronic rapid assessment tool. This was used to assess current and expected capacity at 19 centralized laboratories, as well as to determine what upgrades would be needed at transport hubs, where blood samples for VL from small facilities are centrifuged, to enable samples to be collected from surrounding facilities and transported as plasma to the VL testing laboratories.
3. District Health Information System II (DHIS2) data: routinely reported data provided by the Ministry of Health included the current number of patients on ART at each facility as of March 2017 8. This information was used to derive the estimated sample volumes by facility.

### 2.2 Patient volume estimation

Baseline (current) patient volumes were based on the DHIS2 data at the health facility level from March 2017. To determine the expected increase in volume to meet Zambia's ART coverage and VL monitoring targets, we utilized the following formula:

$$V_{\mathrm{facility}}^{E}=\left({\text{ART}}_{\mathrm{facility}}^{A}*V_{\mathrm{Prov}}^{I}*T^{A}\right)+\left({\text{ART}}_{\mathrm{facility}}^{P}*V_{\mathrm{Prov}}^{I}*T^{P}\right)$$

where $V_{\mathrm{facility}}^{E}$ is the expected (*E*) VL volume in 2020 by facility, ${\text{ART}}_{\mathrm{facility}}^{A}$ is the current number of adult patients on ART by facility, ${\text{ART}}_{\mathrm{facility}}^{P}$ is the current number of paediatric patients on ART by facility, $V_{\mathrm{Prov}}^{I}$ is the expected increase in numbers of patients on ART by 2020 by province based on provincial Spectrum Modelling results 11, $T^{A}$ is the estimated average number of VLs per adult patient on ART per year (1.2) and $T^{P}$ is the estimated average number of VLs per paediatric patient on ART per year 2.

### 2.3 Existing VL testing facilities

VL testing is currently performed at 19 laboratories across Zambia. Current equipment being used includes the Roche Cobas^®^Ampliprep/Cobas^®^TaqMan 48 and the Roche Cobas^®^Ampliprep/Cobas^®^TaqMan 96 (Roche Molecular Diagnostics, Branchburg, US).

All facilities that provide HIV treatment in Zambia are expected to send VL blood samples to one of the laboratories by motorbike, car, truck or using public transport, with the driver/courier employed by the treatment facility itself or by a nongovernmental partner, the laboratory or the district government. VL blood samples need to be centrifuged within six hours of a blood draw, or within twenty‐four hours of a blood draw if refrigerated, in order to retain their integrity.

### 2.4 Healthcare facilities

The 1484 HIV outpatient treatment facilities in our analysis included primary health clinics and hospitals with outpatient services. The estimated >1.2 m patients on ART in 2020 were allocated to these HIV treatment facilities based on each facility's current patient numbers and estimated future 2020 demand. Among HIV treatment facilities, 171 were designated high volume (anticipated ≥10 VLs per day per facility) and represent 76% of total demand. The remaining 1313 facilities were designated low volume (anticipated <10 VLs per day per facility) and represent 24% of total demand. Thresholds for high‐ and low‐volume facilities were selected in consultation with local partners. All high‐volume facilities were prioritized to have VL samples collected daily and transported to the closest VL laboratory. Low‐volume facilities were assigned to weekly sample transport to either hubs or VL laboratories.

### 2.5 Transport hubs

Transport hubs are high‐volume facilities and/or VL laboratories chosen to service surrounding low‐volume facilities on a weekly basis, as well as any high‐volume facilities that are not within a reasonable distance from the VL laboratory. Transport hubs were identified to perform this role and where necessary, centrifuges were allocated to these transport hubs. Transport hubs were selected by utilizing the ArcGIS Network Analyst tool, the Location Allocation solver. The location allocation algorithm uses a heuristic process to solve for a solution where as much VL demand as possible is covered within the specified drive time (120 minutes to ensure ample time to collect samples, visit multiple sites and return to the transport hub or laboratory in the same working day) from the chosen transport hub. Twenty‐six transport hubs were identified and their suitability for acting as transport hubs was validated with in‐country partner and laboratory assessment input. The same transport hubs were used in both scenarios.

### 2.6 Sample transportation scenarios

We evaluated three scenarios for sample transport. First, we estimated the cost per sample transported under the 2017 status quo from a subset of data, and then calculated the expected cost of achieving the same coverage as for the other scenarios for 2020 volumes.

The status quo is used only for comparison, as our goal was to describe two optimized scenarios. Since many healthcare services are organized at the district or provincial level, we defined two optimized scenarios: a district‐bounded scenario and national borderless scenario. In the district‐bounded scenario, all healthcare facilities sent samples to the VL laboratories located in the same province. In the borderless scenario, we ignored district and provincial boundaries and optimized to minimize costs based on the relative locations of laboratories and clinics. Both optimized scenarios were designed to reach the same facilities and VL volumes.

### 2.7 Transport routing

Vehicle routing was determined using an ArcGIS Network Analyst tool that optimized a set of transport routes that took into account expected sample volumes, distance from the VL laboratory or transport hub to the facility and drive times. The optimization algorithm was constrained by practical considerations including service time and driver working hours (Table S1). With these constraints, the tool used a heuristic process to minimize the objective function of reducing travel time and driving distance. The routing algorithms and vehicle routing problem description can be found in Text S1 and Figure S1 as well as a schematic of the flow from low/high‐volume facilities to hubs and laboratories (Figure S2).

### 2.8 Coverage targets

For all three scenarios, we caused coverage of VL testing to increase from 10% in 2016 to 91% in 2020, reaching a total of 800 HIV treatment facilities with an estimated maximum VL volume of approximately 1.5 million samples per year. The coverage target set by the Zambian Ministry of Health was 80% of VL volumes. However, since this target was nearly attained by only routing to high‐ART‐volume facilities, the target for the model was increased, in consultation with local partners, to include additional lower volume facilities in order to provide more equitable access.

### 2.9 Cost analysis

The yearly recurrent costs for operating the STN was calculated in the costing analysis (Table 1). This included vehicle running costs incurred per kilometre of travel (fuel, maintenance and insurance), recurrent vehicle and motorbike capital costs that need to be budgeted for annually as well as personnel costs operating the system (Ministry of Health salaries for drivers as well as additional laboratory assistants). A full costing of a VL processed at a centralized laboratory was conducted in order to put the sample transport cost into perspective. A cost per VL sample transported is then calculated, or the total cost of the STN divided by the estimated number of VLs to be transported. Total annual cost of operating the STN is also reported. Costs are reported in 2018 USD.

**Table 1:** Key cost parameters and related assumptions

Parameter	Estimate	Range	Source
Vehicle running costs ($USD/km)^a^	$0.54	$0.53 to $0.61	Ministry of Health Reimbursement Rate
Diesel price ($USD/litre)	$1.29	$1.21 to $1.38	http://www.globalpetrolprices.com/Zambia/diesel_prices/
Working life of vehicles and motorbikes (years)	4 years	3 to 5 years	Assumption
Recurrent vehicle/motorbike capital costs ($USD)^b^			
Vehicle	$34,778	$30,745 to $37,825	Quotes: 4×4 pick‐up trucks: Toyota Ford Ranger, Mitsubishi, Nissan
Motorbike	$4260	$4248 to $5310	USAID Procurements
Monthly personnel costs ($USD)			
Driver	$400	$300 to $500	Ministry of Health salary scales
Laboratory technologist	$600	–	Ministry of Health salary scales
Per diems ($USD/day)^c^	$17.50	–	Ministry of Health salary scales
Personnel days worked per year	229	200 to 249	Ministry of Health leave days and public holidays for Zambia
Exchange rate (Zambian Kwacha to $USD)^d^	0.100	–	http://www.oanda.com

^a^Zambian reimbursement formula is: ((Fuel Price×1.1)/2.5). ^b^While technically included in the reimbursement formula, these were taken as an additional expense as the reimbursement formula often under budgets for maintenance. ^c^Per diems are calculated for drivers who are away during lunchtime. ^d^Average exchange rate from March to September 2018.

While the optimized STN is based on every HIV treatment facility in the country, the current cost of sample transport is estimated using the results of the EQUIP GIS survey from a subset of facilities. This survey describes whether or not a facility currently transports VLs, type of transport used and the frequency. From facilities that have reported information on collecting and transporting VLs, we calculated the driving distance to the nearest VL laboratory, and assumed that each facility independently transports to the nearest laboratory at the reported frequency. To avoid overestimating the cost, if multiple facilities are located in the same district, and they report using the same mode of transportation, we assumed that they share this vehicle or motorbike. Of these facilities, we then also only used the kilometres travelled to the furthest facility, and not the sum of the kilometres to each facility, to avoid overestimating kilometres driven. When we scaled up the status quo to 2020 volumes and applied the daily/weekly transport requirement to the facilities that reported transporting VLs in 2017, we assumed that there would be no natural improvement of coordination between facilities: that is, if there was no motorbike/vehicle sharing between facilities in 2017, there will be no sharing in 2020.

### 2.10 Sensitivity analysis

To assess the robustness of our model and conclusions, we conducted a multi‐one‐way sensitivity analysis of the key cost inputs. We calculated the change in cost per VL transported in both the borderless and district scenarios for (1) change in Zambian Kwacha to US Dollar exchange rates (±20%), (2) the use of private sector salaries in place of Ministry of Health salaries, (3) the use of either only motorbikes or only vehicles for the whole system, (4) an increase in the price of diesel (+50%), and (5) doubling the expected working life of a vehicle/motorbike.

## 3 Results

We included 19 laboratories, 26 transport hubs and 1484 healthcare facilities in the analysis. As noted above, laboratory location was predetermined by existing infrastructure, and transport hubs were selected from high‐volume facilities and laboratories based on density of and demand from low‐volume facilities within a two‐hour drive time. The full cost of a VL test processed at a centralized laboratory, excluding transport costs, is calculated to be $18.90 (Text S2, Table S2).

In 2017, 726,916 patients were on ART 8, and 476,000 VL tests were conducted (local data; multiple VL tests may be for the same patient). Although there was no formal national blood sample transportation system to serve the 1484 HIV treatment facilities in Zambia, 1178 had data on VL sample transport and just over 10% of those facilities (148/1178) reported having transported at least one VL to a laboratory in 2017 (Table 2). Of those facilities that did transport VL samples, nearly 20% transport samples at most once per month and 16% transport samples at irregular intervals. Just 11% of facilities that transport blood samples transported these samples to laboratories daily, despite the fact that 36% of the facilities are considered high volume.

**Table 2:** Current status of blood‐based viral load sample transportation in Zambia

HIV treatment facility characteristics	Estimate
Has facility transported viral load samples in 2017, n (%) (n=1178)^a^	
Yes	148 (13)
No	1030 (87)
Viral load volume, n (%) (n=148)	
High volume (expected weekly 2020 volumes >10/week)	53 (36)
Low volume (expected weekly 2020 volumes <10/week)	95 (64)
Facility type, n (%) (n=148)	
Level 2 provincial hospital	5 (3)
Level 1 district hospital	44 (30)
Urban health centre	25 (17)
Rural health centre	73 (49)
Health post	1 (1)
Type of transport used, n (%) (n=148)	
Vehicle	94 (63)
Motorbike	44 (30)
Public transport	6 (4)
Viral load done onsite	3 (2)
Boat	1 (1)
Frequency of blood sample transport, n (%), (n=148)	
Daily	16 (11)
Twice weekly	10 (7)
Weekly	59 (40)
Twice monthly	10 (7)
Monthly	28 (19)
Irregular	25 (16)

a Data missing on viral load sample transport from 306 health facilities. These 306 health facilities were distributed across all 10 provinces.

The status quo cost per sample transported was estimated at $9.92 (SD $1.25), or 34% of the cost of a VL processed in a centralized laboratory. This equates to a total annual cost of $1.36 m for these 148 facilities served (Table 3). Cost was high due to low demand for VL, inefficient routing and limited sharing of transport between facilities within the same vicinity. When we expanded the status quo to match the demand for VL testing (the expected demand in 2020 due to growth in number on ART and increased VL demand in these 148 facilities) and frequency of transport assumed in our model (either increased to daily or weekly sample collection), the status quo cost per sample transported fell only marginally to $9.54 (SD $1.22) or 34% of the cost of a VL. This equates to $4.3 m (SD $547,000) annually for these facilities.

**Table 3:** Cost of the status quo from a subset of facilities that reported transporting viral loads in 2017

Parameter	Status quo 2017	Status quo in 2020^a^
Facilities reached	148	148
Total weekly viral load volumes	2635	8588
Total weekly kilometres driven	32,200	100,000
Number of full‐time equivalent vehicles used/required^b^	18	61
Number of full‐time equivalent motorbikes used/required^b^	8	21
Number of full‐time equivalent drivers^b^	26	82
Efficiency (kilometres driven per viral load)	12.2	11.6
Transportation cost per viral load	$9.92 (SD $1.25)	$9.54 (SD $1.22)
Total annual cost	$1.36 m (SD $171,000)	$4.3 m (SD $547,000)

^a^Using currently available transport types and coordination only improved to reach high‐volume facilities daily and low‐volume facilities weekly. ^b^Most vehicles/drivers are not full‐time given the infrequency of transport.

Under both optimized STNs, a total of 152 high‐volume facilities were reached *daily*. These 152 facilities represent 70% of the total anticipated 2020 sample volume. An additional 637 low‐volume facilities plus 11 high‐volume facilities were reached *weekly* under both optimized STNs. These 648 facilities represent 21% of the anticipated 2020 sample volume (6800 weekly samples).

**Figure 1:**
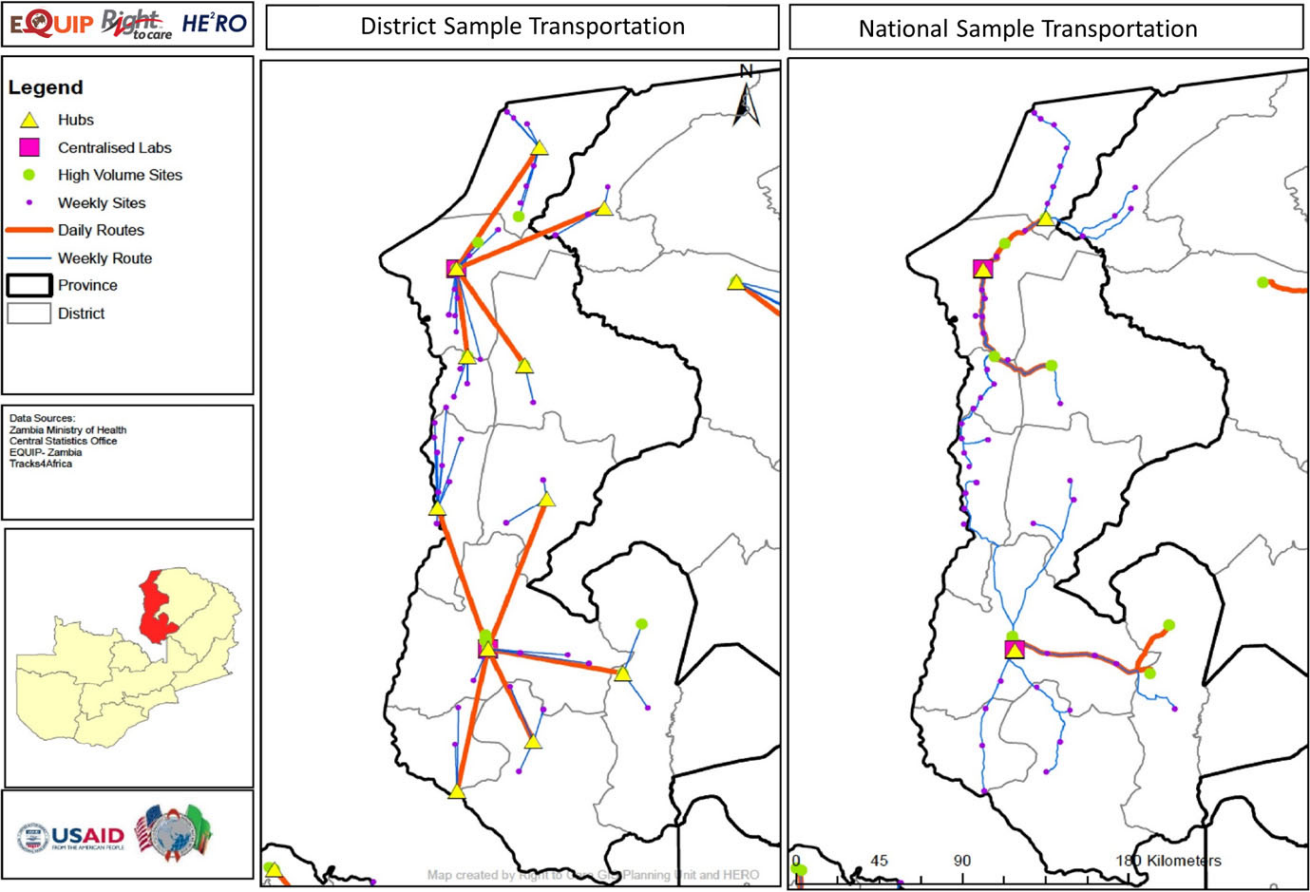
District‐bounded versus national borderless sample transportation network: illustrating daily and weekly vehicle routing between high‐volume and low‐volume facilities to hubs and viral load centralized laboratories This map shows the facilities reached and routes taken by the STNs. Pink squares represent viral load laboratories, yellow triangles represent chosen hubs, orange lines represent daily transport routes and blue lines represent weekly transport routes. The district‐bounded approach utilizes more transport hubs (one per district) and as such requires additional routing to laboratories. STN, sample transportation networks.

Although both optimized scenarios were modelled to reach the same coverage targets, the distance and resources required to reach these facilities differed sharply (Table 4). Under the borderless scenario, a total of 57 vehicles and 13 motorbikes were required to sustain the system, while the district‐bounded scenario required 111 vehicles and 162 motorbikes. The total distance travelled under the district‐bounded STN was 58% more than the borderless STN at 96,849 km versus 61,111 km for national coverage. This corresponds to a higher number of kilometres driven per VL for the district‐bounded STN (3.24) versus the borderless STN (2.00). Figure 1 illustrates the vehicle routing for Zambia's Luapula Province between high‐volume facilities and centralized laboratories and transport hubs for both the borderless and district‐bounded scenarios. Similar routing was conducted for all ten provinces.

**Table 4:** Result summary of the optimized borderless and district‐bounded sample transportation network

Parameter	Borderless	District‐bounded
Number high‐volume facilities reached	163	163
Number low‐volume facilities reached	637	637
Total number of facilities reached	800	800
Total weekly viral load volumes at high‐volume sites	22,993	22,993
Total weekly viral load volumes at low‐volume sites	6850	6850
Total weekly viral load volumes	29,843	29,843
Total weekly kilometres driven	61,111	96,849
Number of vehicles required	57	111
Number of motorbikes required	13	162
Number of drivers	70	273
Efficiency (kilometresdriven per viral load)	2.0	3.24
Transportation cost per viral load		
High‐volume facilities	$1.74 (SD $0.17)	$2. 55 (SD $0.29)
Low‐volume facilities	$3.33 (SD $0.43)	$10.47 (SD $1.51)
Total system	$2.11 (SD $0.28)	$4.37 (SD $0. 69)
Total annual cost	$3,265,000 (SD $431,000)	$6,782,000 (SD $1,078,000)

The greater efficiency of the borderless scenario is reflected in the costs. The mean transport cost per VL sample transported in the borderless STN was $2.11 per test (SD $0.28), 52% less than the mean cost per sample transported in the district‐bounded STN of $4.37 (SD $0.69), comprising 10% and 19% of the cost of a VL respectively. Nationwide, the district‐bounded STN would cost an average of $6,782,000 (SD $1,078,000) per year while the borderless STN would cost an average of $3,265,000 (SD $431,000). When fully scaled‐up to the anticipated 2020 volumes, the borderless system would thus save the government of Zambia $3,479,000 (SD $647,000) per year on sample transportation. This equates to approximately 2.6% of the total current cost of the Zambian national ART programme in the district‐bounded STN compared, and 1.2% of the current cost of the Zambian national ART programme in the borderless STN 12. The cost per sample transported under the optimized district‐bounded scenario is less than half of that found under the status quo scaled up to 2020 volumes ($4.37 vs. $9.54) when the demand and frequency of transport is matched to that of our model, showing the value of organizing a transport system.

**Figure 2:**
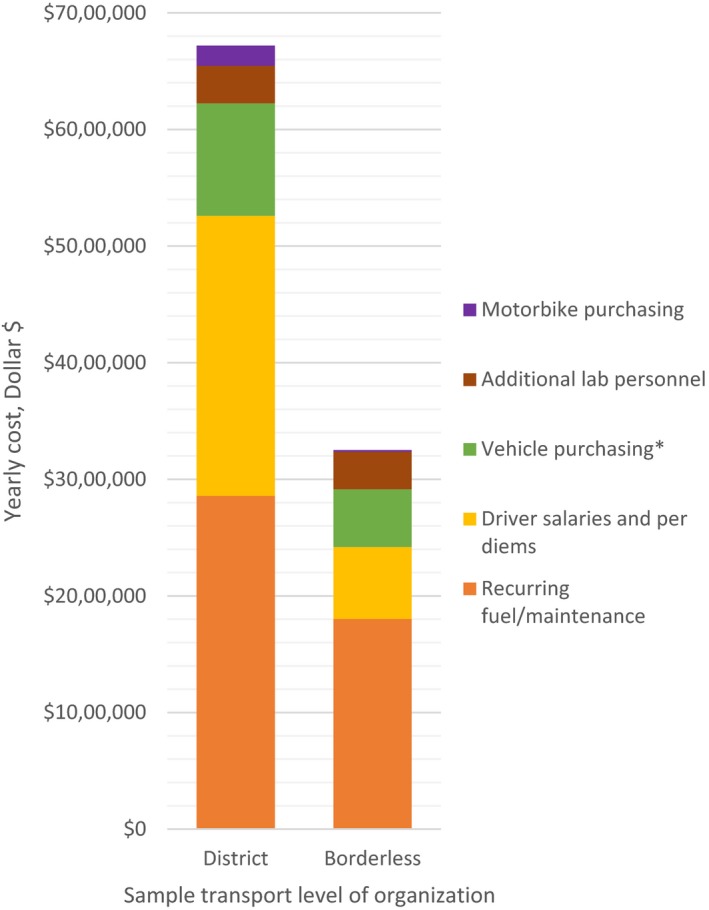
Recurrent annual cost breakdown between district‐bounded and borderless sample transportation network scenarios *Cost of vehicle and motorbike purchasing annualized over four years.

This savings is primarily due to a reduction in the number of vehicles and drivers needed, along with more efficient routes enabled by interdistrict routing. The primary source of the cost difference between the two scenarios are driver salaries and per diems: from $2,402,000 (SD $444,000) in the district‐bounded scenario to $616,000 (SD $114,000) in the borderless scenario, followed by the recurrent fuel/maintenance cost: from $2,858,000 (SD $199,000) in the district‐bounded scenario to $1,803,000 (SD $126,000) in the borderless scenario (Figure 2).

There are relatively more savings in cost per sample transported from the low‐volume clinics than from the high‐volume clinics. For low‐volume facilities, the cost per sample was $3.33 (SD $0.43) in the borderless scenario compared to $10.47 (SD $1.51) in the district‐bounded scenario. For high‐volume clinics, these figures were $1.74 (SD $0.17) compared to $2.55 (SD $0.29) respectively (Table 4). A borderless STN will save 32% of costs for high‐volume sites and 68% of costs for low‐volume sites, compared to a district‐based STN. This results from the fact that in the district‐bounded scenario, all 109 districts in Zambia require at least one vehicle or motorbike (and driver) to be available, regardless of demand and number of facilities in a district, a condition that can be relaxed in the borderless scenario, allowing more room for savings.

One‐way sensitivity analyses (Table S3) highlight five key cost input parameters of our model. The model is most sensitive to (1) a decrease in the Zambian kwacha:US dollar exchange rate by 20%, resulting in a 20% decrease in the cost per test transported in both district and borderless scenarios and (2) an increase in the price of diesel (+50%), resulting in a 17% increase in the cost per test transported in the district scenario and a 22% increase in the borderless scenario. The magnitude of difference between the district and borderless scenarios was, however, stable: a minimum difference of 49% (with increase in diesel price) and a maximum difference of 58% (when only vehicles are used in the transport system).

## 4 Discussion

Using a newly developed geospatial optimization model, we calculated that a VL blood sample transport network that is borderless – does not take into account political boundaries within Zambia – has the potential to lower costs by 52% compared to a STN that is optimized within district‐level boundaries. This provides a rare opportunity for increased efficiency in an era where cost savings, particularly in HIV care, are increasingly difficult to come by. Sample transport currently comprises 34% of the total cost of a VL test, and threatens to increase substantially as countries scale up test access to hard‐to‐reach populations. By optimizing the transport network, the additional costs that countries and funders will incur for scaling up VL testing to achieve targets can be partly offset by greater efficiency in transport. This is apparent given more than an annual $1.36 m is being spent just to reach 148 facilities, representing just a quarter of all VLs reached by the optimized STN, and only collected samples less than once per week for 42% of those facilities. Reaching all facilities covered by our model, at the desired frequency of services, will cost well over the estimated costs for both the district and borderless scenarios.

As noted, we only had transport information on a subset of facilities to estimate the status quo. We believe that this is likely to underestimate of the cost per test nationally. The facilities included in the status quo analysis were more likely to be high volume than in our STN (36% vs. 19%) and thus should be less expensive to reach per sample transported due to high volumes.

The costs and cost per sample reported here, for all scenarios, are for a dedicated VL sample transport network. The incorporation of other types of samples into this network will significantly reduce the total cost per VL sample transported if each specimen shares part of the cost and will improve overall clinical services by making a range of laboratory tests more accessible.

Geospatial modelling in the HIV field has been used primarily to predict incidence and the impact of treatment and prevention on incidence 13, 14, 15, 16, or to inform targeted interventions 17, 18, 19. To our knowledge, this is the first programmatic geospatial model created to optimize an STN and to examine the differences between the levels of organization of an STN, whether organized at the district or national level. It is also the first geospatial model that attempts to describe ideal VL scale‐up strategies based on entirely local data, and to geographically integrate multiple levels of information – facility infrastructure, laboratory infrastructure, local epidemiological data and road network/accessibility data – to provide practical programmatic guidance.

There are several limitations to this approach. First, two VLs per paediatric patient per year and 1.2 VLs per adult per year may overestimate the number of VL testing actually done per year even when access improves. Similarly, the number of patients on ART may not reach the number of patients estimated by the Ministry of Health and Spectrum Modelling results. Ideal patient volumes were chosen deliberately to plan for the maximum possible number of VLs in the system. Planning for a more realistic number of VLs would change the magnitude of the annual cost of the STN but would not change the district‐based versus borderless STN scenarios differentially. Second, we assumed that the equipment at existing VL laboratories can be updated or increased to cope with the volumes predicted in our model. That said, it will realistically take a number of years to implement the full STN, allowing time for the government and implementing partners to scale up laboratory infrastructure accordingly. All the laboratories used in the analysis have the capacity to be upgraded to higher capacity equipment. Third, weekly sample collection at low‐volume facilities would mean having only one designated day of the week for drawing blood. While this could increase the required number of clinic visits for some patients, many low‐volume facilities already designate an “ART” day in which all ART‐related services are provided only one day per week. We have not explored how the use of dried blood specimens would allow these low‐volume facilities to draw samples more frequently and transport less frequently, as dried specimens for VL have not been approved for use in Zambia. Fourth, this model aims to reach at least 80% patient volumes, in line with the goals of the Zambian government, and assumes that only centralized laboratory testing will continue to be used. The model therefore prioritizes high‐volume clinics and does not account for the possibility of point‐of‐care VL testing. Fifth, implementation of a borderless STN requires coordination across all partners and between districts and provinces that do not traditionally collaborate. While this will be challenging to achieve at the start, we hope that the potential savings and interest in the long‐term sustainability of the programme will motivate all players to work together. Sixth, while we have reported optimal transport scenarios, it is important to note that the scenarios are not optimized on costs specifically. Within ArcGIS, we have used the tools to minimize time and distance travelled while maximizing volume, we have then assumed that this applies directly to related costs. It is possible that if a different approach or geospatial program were used, that more optimal routes could be found. This may affect our point estimates of total cost and cost per sample transported, but is unlikely to affect the magnitude of difference between scenarios. Finally, our road network was based on driving times collected in the wet season when road conditions are at their worst. As a result, driving times might be overestimated, increasing the overall estimated costs of the STN for both the district‐bounded and borderless scenarios.

The results of this analysis and the reported potential for cost savings are generalizable across a broad number of countries that currently utilize district‐ or provincial‐based planning for sample transportation, including South Africa, Mozambique, Uganda, Zimbabwe and others 5. This methodology could also be adapted to improve laboratory access for other conditions than HIV, achieve optimal placement of equipment and evaluate trade‐offs between centralized and decentralized or point‐of‐care laboratory testing.

## 5 Conclusions

To conclude, we found that an efficient STN that optimizes sample transport on the basis of geography and test volume, rather than political boundaries, can cut the cost of sample transport by more than half in Zambia. This model, which can be used in other countries and for other types of samples, has the potential to increase the sustainability of ART programmes throughout Africa.

## Competing interests

The authors declare that no competing interests exist.

## Authors’ contributions

BEN, CM and JK conceived the study. BEN, SJG, TC, LS‐I, LB, DC and WS acquired and analysed data for the model. BEN, SJG and TC developed the model. BEN and SR interpreted the model results. BEN, SJG and SB wrote the first draft of the manuscript. BEN, SJG, TC, LS‐I, LB, DC, CM, JK, WS and SR contributed to the writing of the manuscript. BEN, SJG, TC, LS‐I, LB, DC, CM, JK, WS and SR read and approved the final manuscript.

## Supporting information


**Text S1.** Vehicle routing.
**Text S2.** Viral load costing.
**Table S1.** Key vehicle routing modelling assumptions.
**Table S2.** Centralized viral load cost per test – assumptions and sources.
**Table S3.** Sensitivity analysis: one‐way sensitivity analysis of key cost input parameters.
**Figure S1.** Vehicle routing problem in model builder for high‐volume facilities.
**Figure S2.** Schematic of the simplified transportation network.
