# Supplementary material for: Impact of a borderless sample transport network for scaling up viral load monitoring: results of a geospatial optimization model for Zambia
Source: J Int AIDS Soc. 2018 Dec 4;21(12):e25206. doi: 10.1002/jia2.25206 (PMC6280013; doi:10.1002/jia2.25206)
Supplement: Supplementary file 1 — Text S1. Vehicle routing. Text S2. Viral load costing. Table S1. Key vehicle routing modelling assumptions. Table S2. Centralized viral load cost per test – assumptions and sources. Table S3. Sensitivity analysis: one‐way sensitivity analysis of key cost input parameters. Figure S1. Vehicle routing problem in model builder for high‐volume facilities. Figure S2. Schematic of the simplified transportation network. [file JIA2-21-e25206-s001.docx]

**Supplementary Appendix**

**Text S1. Vehicle Routing**

Two vehicle routing approaches were identified: (1) daily sample collection (from high volume facilities to the centralized labs) and; (2) weekly sample collection (from low volume facilities to the transport centers). The two vehicle routing problems were solved using an ArcGIS Network Analyst tool which optimizes a set of routes taking into account expected sample volumes, distance from the lab or transport center to the facility, and drive times. In addition, the optimization algorithm was constrained by practical considerations including service time and driver working hours (Table S1). No capacity constraints were place on our routes as we assumed that centralized viral load labs would scale up sufficiently to meet the viral load demand.

**Table S1. Key vehicle routing modelling assumptions**

| **Modelling Assumptions** | **Description** | **Unit Estimate** |
| --- | --- | --- |
| Capacity at centralised viral load labs | Centralised labs can be scaled up sufficiently to meet the expected viral load demand | N/A |
| Sample transport operations | Whilst centralised labs might be operational for more than 5 days a week, we implicitly included a buffer for vehicle break downs or other practical challenges that might prevent drivers from operating seamlessly every day. | *5 days/ week* |
| Service time | This is the time it takes for the driver to stop the vehicle and collect or deliver the sample before getting back on the road. It was assumed the driver needs 30 minutes at the start of the day, and at the end of the day, as well as 30 minutes at each facility. This adds an additional buffer into the transport system for any unforeseen circumstances that could prevent the system from operating optimally, for example traffic. | *30 minutes* |
| Driver working hours | We have set this constraint in the model such that a driver cannot be on the road, at lunch or collecting and delivering samples for more than 8 hours a day. This is current practice | *8 hours/ day* |
| Driver working day flexibility | Drivers’ starting times can be staggered to accommodate facilities’ needs, and blood sample integrity. |  |
| Driver lunch break | Included in the 8 hour working day. This is current practice | *1 hour* |
| Blood collection at low volume facilities | It was assumed that low volume facilities would organise their ART days (and blood collection days) taking into account the time and day on which their samples are scheduled to be collected by the driver. Assumption based on discussion with in-country implementing partners. | Once a week |
| Vehicles and motorbikes are based at the assigned lab or transport centre | This ensures that the lab and transport centres are ultimately accountable for the vehicles assigned to them and for operating the routes. | N/A |
| Motorbikes are suitable for routes of less than 100km. | Routes that are less than 100km are assigned to motorbikes. Routes longer than this are assumed to be more suitable for vehicles given road conditions and wear and tear. Assumption based on discussion with in-country implementing partners. | *100 km* |
| Every hub is assigned one vehicle or motorbike, and are provided with more if the efficiency of the additional routes warrants this. | A route’s efficiency was defined as the number of km required to collect one viral load. If the routes across a week averaged less than 8 km/viral load, an additional vehicle was assigned. Whilst efficiency was taken into account, emphasis was placed on maximising national coverage. As such, each hub was assigned at least one vehicle even when this was at the expense of efficiency. | N/A |
| District based assumptions | District hub status was assigned to viral load labs or the highest viral load volume facility in the district if no lab was present. | N/A |

The vehicle routing problem algorithm solves the routing problem taking into account these constraints while using a heuristic process to minimize the objective function of reducing travel time and driving distance. The solver for the vehicle routing problem first generates an origin-destination matrix of shortest-path driving times between all facilities and all centralized labs using a road network. This cost-matrix is then used to develop an initial solution by allocating facilities one at a time to the most appropriate route. This solution is then improved by resequencing the facilities allocated to each route, as well as reallocating some facilities to other routes, and exchanging some facilities between routes. The heuristic used in this process is based on ESRI’s proprietary tabu search metaheuristic(1).

The tabu search metaheuristic, used by ESRI, is a methodology that can solve the travelling sales man problem, analogous to the vehicle routing problem. This is a NP-hard, combinatorial optimization problem. The tabu search metaheuristic is the most widely used procedure to solve combinatorial optimization problems(2) It starts with a complete feasible solution and iteratively generates improved solutions. It is unclear whether the initial solution is determined deterministically or randomly (not specified by ESRI). It will stop once a pre-defined number of iterations has been reached (not specified by ESRI) or no improvement is achieved.

We used Model Builder to run the vehicle routing problem Network Analyst tool in ArcGIS. Model Builder is an application in ArcGIS that is used to create, edit, and manage models. Models are workflows that string together sequences of geoprocessing tools feeding the output of one tool into another tool as input. Figure S1 and related description detail the vehicle routing process to high volume facilities from centralized laboratories.

**Figure S1: Vehicle Routing Problem in Model Builder for high volume facilities**


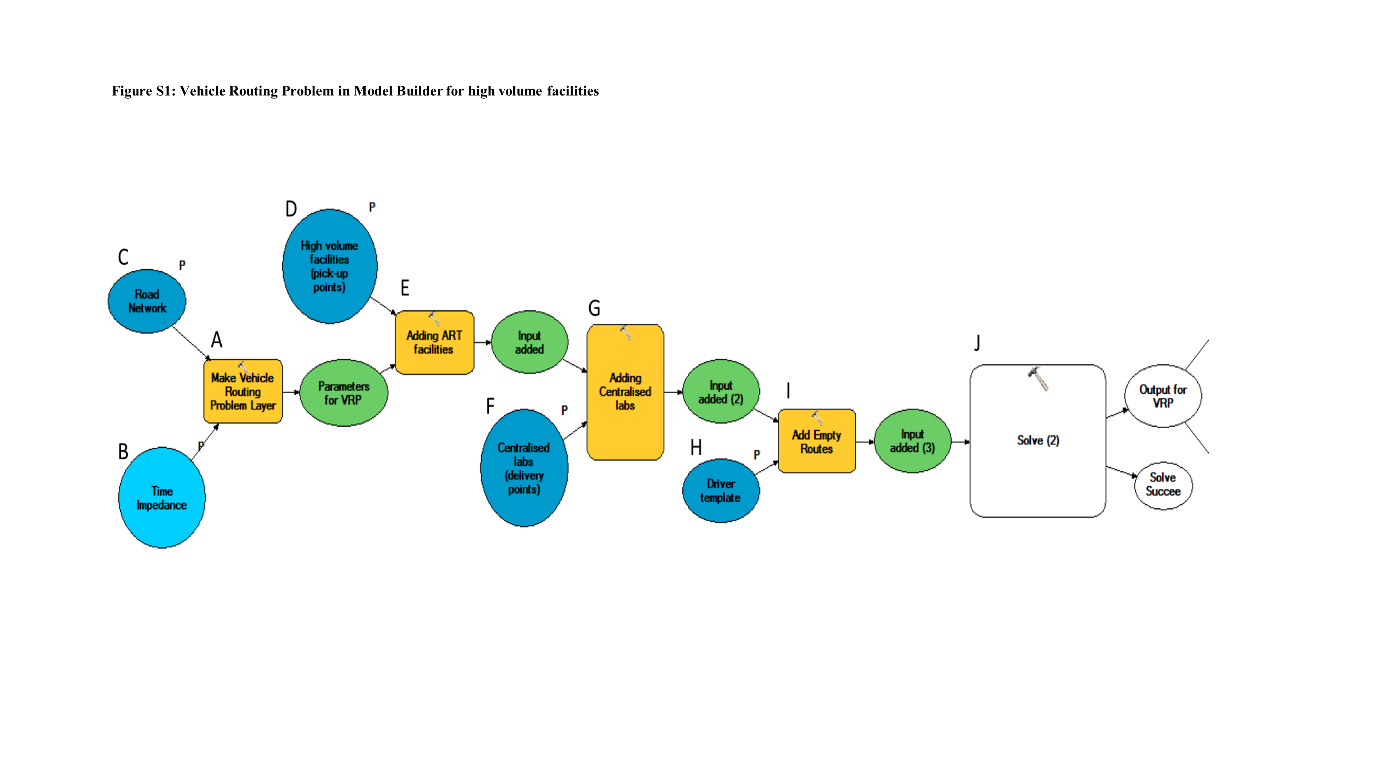


*Figure S1 description:* The process starts with setting up the parameters for the vehicle routing problem (*A*), namely, indicating the impendence factor (or what the solution is constrained by) is driving time (*B*). The road network variable is the input that provides this impedance factor (*C*). We then added the locations of the high volume ART facilities and indicated that at each ART facility 30 minutes’ service time is required and that the daily viral load volumes are the required pick up quantities (*D, E*). We then added the location of the centralized labs – the required start and end points for the vehicle routes (*F, G*). Lastly, we detailed the required driver parameters: drivers can work a maximum of 8 hours a day, including an hour lunch break, and that it takes 30min at the beginning of the shift and again at the end of the shift to load and offload samples at the centralized lab (*H, I*). The solver then generated the origin-destination matrix and applied the heuristic algorithm to generate routes to ART facilities that minimized driving times (*J*). Each centralized lab or hub was initially assigned one driver or route, however, if the route was considered efficient (defined as < 8km/viral load), more routes were assigned to that centralized lab or hub (see Table S1).

This process was repeated for low volume facilities on the weekly routes.

**Figure S2.** Schematic of the simplified transportation network


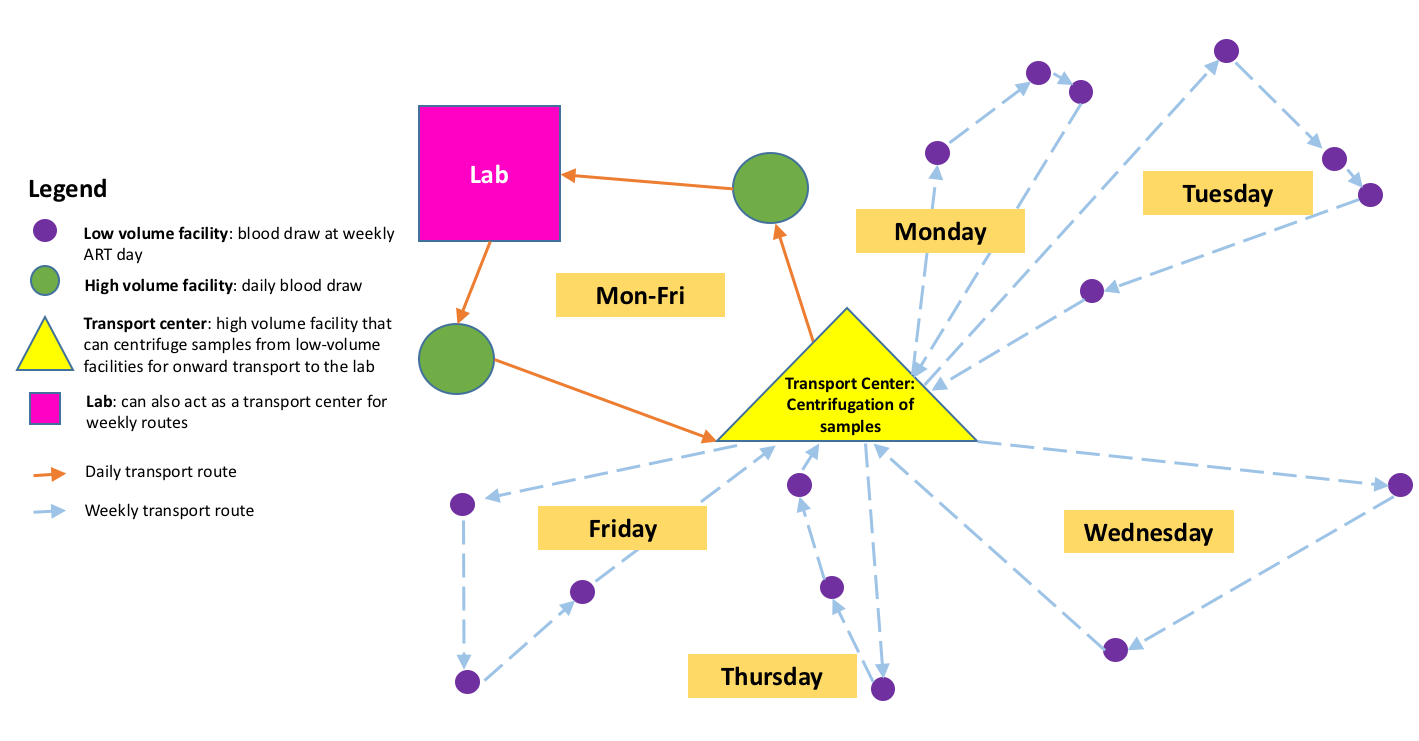


**Text S2. Viral load costing**

The cost per viral load test in a centralized test setting was estimated using the Testing Platform Cost Model (TPCM) developed by HE2RO staff ([www.heroza.org/tools](http://www.heroza.org/tools)). This cost model provides a simple tool for analysts and decision-makers to determine the cost per test for a testing platform from a provider’s perspective. The cost to a provider of a test depends on many individual components. These include the material costs per test, the salaries of staff who conduct start-up and daily quality control activities on the machine, the salaries of staff who perform the test, how the technology is integrated into patient flow and management, equipment and other related costs (e.g. insurance, replacement parts), the expected working life of the platform itself, and the opportunity cost of funds used to acquire the platform (i.e. the discount rate). Shared costs are allocated according to the proportion of lab tests that the test under evaluation accounts for. Overhead costs are allocated according to the space allocated to the specific test under evaluation relative to the entire facility.

For this paper, we based our cost estimates for a viral load conducted at a centralized laboratory on a bottom-up costing analysis conducted at the Centre for Infectious Disease Research in Zambia (CIDRZ) laboratory located in the Kalingalinga area of Lusaka, Zambia. It is the primary laboratory for most of Lusaka province and it serves as a reference laboratory for molecular diagnosis (HIV, DNA, PCR and viral load testing) for a number of neighboring provinces.  It processes and conducts testing on approximately 1600 blood samples per day (of which 400 are viral loads). Viral load testing is conducted using the two Roche Cobas®Ampliprep/ Cobas®TaqMan 96 (CAP/CTM 96) (Roche Molecular Diagnostics, Branchburg, US). There are a number of shared activities across these blood sample tests as all blood samples are sorted, registered and stored together – as such all shared costs from these activities was allocated across all blood samples. We have also costed the viral load sample collection, including the materials, staff-time and overhead required. Costs reported are an average across eight facilities in Lusaka and Central provinces.

The inflation rate was based on data from the Zambian Central statistical Office (Zambia Data Portal) and the exchange rate between the Zambian kwacha and the United States dollar was based on data from the Bank of Zambia. All costs are reported in US dollars and are for 2018.

All assumptions and sources are detailed in **Table S2** below.

**Table S2: Centralized viral load cost per test – assumptions and sources**

| **Cost category** | **Unit cost (US$)** | **Items included** | **Assumptions** | **Sources** |
| --- | --- | --- | --- | --- |
| **Cost of viral load at laboratory** | | | | |
| Materials | 14.74 | Pipette tips; Reagents (including SPU for cobas, Tip-k for cobas ampliprep, S-tube input for cobas, KIT CAP-G/CTM wash reagent) | Included the central Medical Stores Limited distribution margin of 1.5%. | Biogroup Zambia Limited and Medical Stores Limited Catalogue 2016 |
| Staff | 0.47 | Laboratory staff activities for viral load testing included: registering the sample/data entry; sample preparation and running; interpretation of the result/result delivery. Other shared activities conducted by lab assistants on all blood samples included sample sorting, centrifuging, aliquoting, archiving, searching temperature monitoring etc. | Activity time estimations based on discussions with staff at the CIDRZ laboratory as well as lab records. Shared staff time allocated across all blood sample tests. | Government of Zambia salary scales.  CIDRZ laboratory, Lusaka |
| Quality control | 0.04 | Quality control activities conducted by a laboratory scientist every shift included start-up/equipment maintenance (including checking the buffer and control tubes, emptying waste). A cleaner also cleaned the room containing the viral load equipment every day. | Activity time estimations based on discussions with staff at the CIDRZ laboratory | Used Zambian government salaries for staff time. Medical Stores Limited Catalogue 2016; CIDRZ laboratory, Lusaka |
| Equipment | 0.88 | CAP/CTM 96 ($160,000), air-conditioners, refrigerators, fire extinguishers, computers, biosafety cabinets, chairs, freezers, tables generator, thermomixer, printers, uninterrupted power supply etc. | Estimated the working life of a CAP/CTM 96 (5 years); Estimated other equipment working life based on the South African Revenue Service write-off periods as well as discussed with lab personnel. All equipment annualized at 5% discount rate. Shared equipment for the lab allocated across all blood sample tests. | USAID procurement invoices, other invoices, CIDRZ lab finance |
| Other | 0.74 | Includes annual and upfront training for lab personnel; maintenance and insurance costs for equipment (e.g. CAP/CTM 96 and fridges/freezers), waste removal, and dinner and transport allowance for extra shift work. | Viral loads contributes 40% of all lab waste (based on CIDRZ staff estimate) | Used Zambian government salaries for staff time for training, used invoices from CIDRZ lab for maintenance, insurance, staff per diems, and waste removal |
| Overhead | 0.35 | Electricity, security services, motor vehicles, overhead staff (e.g. other laboratory scientists, managers and maintenance staff) | Estimated electricity based on EQUIP office electricity bill. Allocated 20.4% of the total building size to viral load testing activities (based on space used to conduct the test as well as a proportion of shared space). | Discussions with the CIDRZ laboratory senior staff. |
| *Lab-based sub-total* | *17.22* |  |  |  |
| **Cost of sample collection** | | | | |
| Materials | 0.31 | Spirits, cotton wool, syringe, needles, EDTA tubes, specimen bags, globes, specimen containers, apron, waste bin liners, sharps container, chlorine | 5ml of spirit per patient, 0,5g cotton wool per patient, 2 waste bin liners are used per week, 1 apron per nurse per day, 1 disposable sharp box a day, 1 bottle of bleach a week for cleaning. These costs were allocated according to estimated numbers of patients seen for bloods a week. | Zambian Medical Stores Limited Catalogue 2016, and where prices not listed, equivalent material cost obtained through invoices and online quotes. |
| Staff | 0.87 | Nursing staff activities included drawing and collecting blood in an EDTA container. A cleaner’s time for cleaning surfaces and the room was also included. | Activity time estimations based on discussions with staff at CIDRZ lab (12.5 minutes per blood draw). | Government of Zambia salary scales.  CIDRZ laboratory, Lusaka, Zambia. |
| Overhead and Equipment costs | 0.001 | Overheads includes electricity, water, impressed government grant. Equipment includes desk, chair, specimen cooler box, dust bins, ice packs, lab log book, bathroom basin with faucet, tourniquet kit | Overheads allocated according to size of the blood draw room and then allocated according to the number of ART patient visits per month. Equipment allocated according to the number of ART patient visits per month. | CIDRZ laboratory, Lusaka, Zambia. Invoices and quotes. |
| *Sample collection sub-total* | *1.18* |  |  |  |
| **Total** | **18.40** |  |  |  |

| **Table S3. Sensitivity Analysis**: one-way sensitivity analysis of key cost input parameters | | | |
| --- | --- | --- | --- |
|  | *Cost per test transported* | |  |
| **Parameter** | **District-based** | **Borderless** | **Percent saved by adopting borderless vs district** |
| Primary analysis | $4.37 | $2.11 | 52% |
| Decrease in exchange rate (-20%) | $3.65 | $1.76 | 52% |
| Increase in exchange rate (+20%) | $5.09 | $2.46 | 52% |
| Private sector salaries used | $4.46 | $2.13 | 52% |
| Only motorbikes used to transport samples | $3.80 | $1.82 | 52% |
| Only vehicles used to transport samples | $5.20 | $2.18 | 58% |
| Diesel price increase (+50%) | $5.29 | $2.69 | 49% |
| Double expected working life of vehicle/motorbike | $3.98 | $1.94 | 51% |

**References**

1. ESRI. Algorithms used by network analyst [Internet]. [cited 2018 Sep 11]. Available from: http://desktop.arcgis.com/en/arcmap/latest/extensions/network-analyst/algorithms-used-by-network-analyst.htm

2. Basu S. Tabu Search Implementation on Traveling Salesman Problem and Its Variations: A Literature Survey. Am J Oper Res [Internet]. 2012;02(02):163–73. Available from: http://www.scirp.org/journal/doi.aspx?DOI=10.4236/ajor.2012.22019
